# Derivatization enhances analysis of estrogens and their bioactive metabolites in human plasma by liquid chromatography tandem mass spectrometry

**DOI:** 10.1016/j.aca.2018.12.023

**Published:** 2019-04-25

**Authors:** Nina Denver, Shazia Khan, Ioannis Stasinopoulos, Colin Church, Natalie ZM. Homer, Margaret R. MacLean, Ruth Andrew

**Affiliations:** a Mass Spectrometry Core, Edinburgh Clinical Research Facility, Queen's Medical Research Institute, 47 Little France Crescent, Edinburgh, EH16 4TJ, United Kingdom; b Institute of Cardiovascular and Medical Sciences, College of Medical, Veterinary and Life Sciences, University of Glasgow, University Avenue, Glasgow, G12 8QQ, United Kingdom; c Scottish Pulmonary Vascular Unit, Golden Jubilee National Hospital, Agamemnon St, Clydebank, G81 4DY, United Kingdom

**Keywords:** Derivatization, Estrogen, Liquid chromatography tandem mass spectrometry, Estrogen metabolites, Methylpiperazine, CID, collision-induced dissociation, cps, counts per second, E1, estrone, OHE1/2, hydroxyestr-one/adiol, MeOE1/2, methoxyestr-one/adiol, ESI, electrospray ionization, FA, formic acid, IS, internal standards, LC-MS, liquid chromatography-mass spectrometry, LC-MS/MS, liquid chromatography tandem mass spectrometry, LODs, limits of detection, LLOQ/ULOQs, lower/upper limits of quantitation, PPZ, 1-(5-fluoro-2,4-dinitrophenyl)-4-methylpiperazine, MPPZ, methyl-PPZ-, MRM, multiple reaction monitoring, PAH, pulmonary arterial hypertension, RME, relative mean error, RSD, relative standard deviation, SD, standard deviation, SNR, signal/noise, SPE, solid phase extraction

## Abstract

Estrogens regulate many diverse biological processes in health and disease. They circulate at a wide range of concentrations in females generating several active metabolites (hydroxy and methoxyestrogens). The metabolites are assumed to be present in much lower levels and are thought to contribute to diseases such as pulmonary arterial hypertension (PAH). Estrogen metabolites are challenging to quantify in plasma and currently available immunoassays are non-specific. Here we have developed and validated a novel assay to simultaneously quantify parent estrogens and their metabolites by mass spectrometry (MS).

Estrogens were extracted from human plasma using solid phase extraction and derivatized using 1-(5-fluoro-2, 4-dinitrophenyl)-4-methylpiperazine (PPZ) before quaternization by methylation (“MPPZ”). MPPZ derivatives were separated and quantified by liquid chromatography tandem MS (LC-MS/MS) in positive electrospray ionization mode, using a QTrap 6500 + coupled to a Shimadzu Nexera X2. Separation was achieved using an ACE Excel 2 C18-PFP column (2 μm, 2.1 mm × 150 mm). The limits of quantification (LOQ) were 0.43–2.17 pg on column with a linear range from 2 or 10 - 2000 pg mL^-1^. Intra and inter-day precision and accuracy were acceptable (<20% at LOQ and <15% above). These derivatives demonstrated minimal degradation upon short-term storage at 15 °C (<20%) and longer term at −20 °C (<20%). Using this approach, estrone (E1) and estradiol (E2) were detected in plasma (0.5 mL) from healthy women and those with PAH but downstream metabolites 16-hydroxy-E1, 16-hydroxy-E2, 2-methoxy-E1 and 4-methoxy-E1 were only detected in plasma from diseased patients. These findings will next be tested robustly in large patient cohorts.

This novel LC-MS/MS analysis of estrogens and their bioactive metabolites, using MPPZ derivatization, opens doors for the simultaneous analysis of a panel of estrogens in human plasma, across the endogenous range of concentrations encountered in health and disease.

## Highlights

• LC-MS/MS method for simultaneous quantification of estrogens and their bioactive metabolites in human plasma.
• High recoveries and reduced matrix effects using MCX-SPE^®^ extraction cartridges followed by MPPZ derivatization.
• Generation of stable derivatives allowing quantification of estrogens in low endogenous levels.

## Graphical abstract

**Figure:**
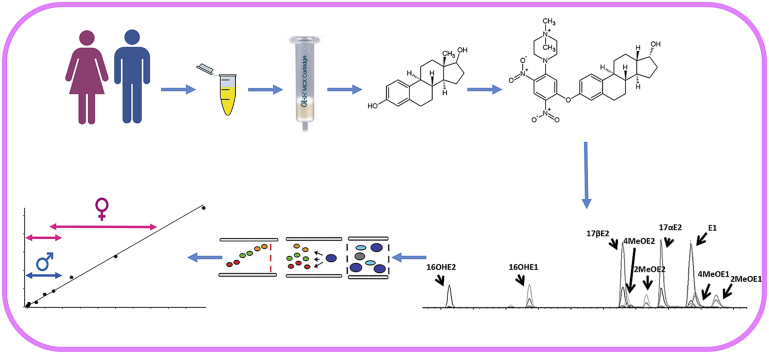

## 1 Introduction

**Fig. 1:**
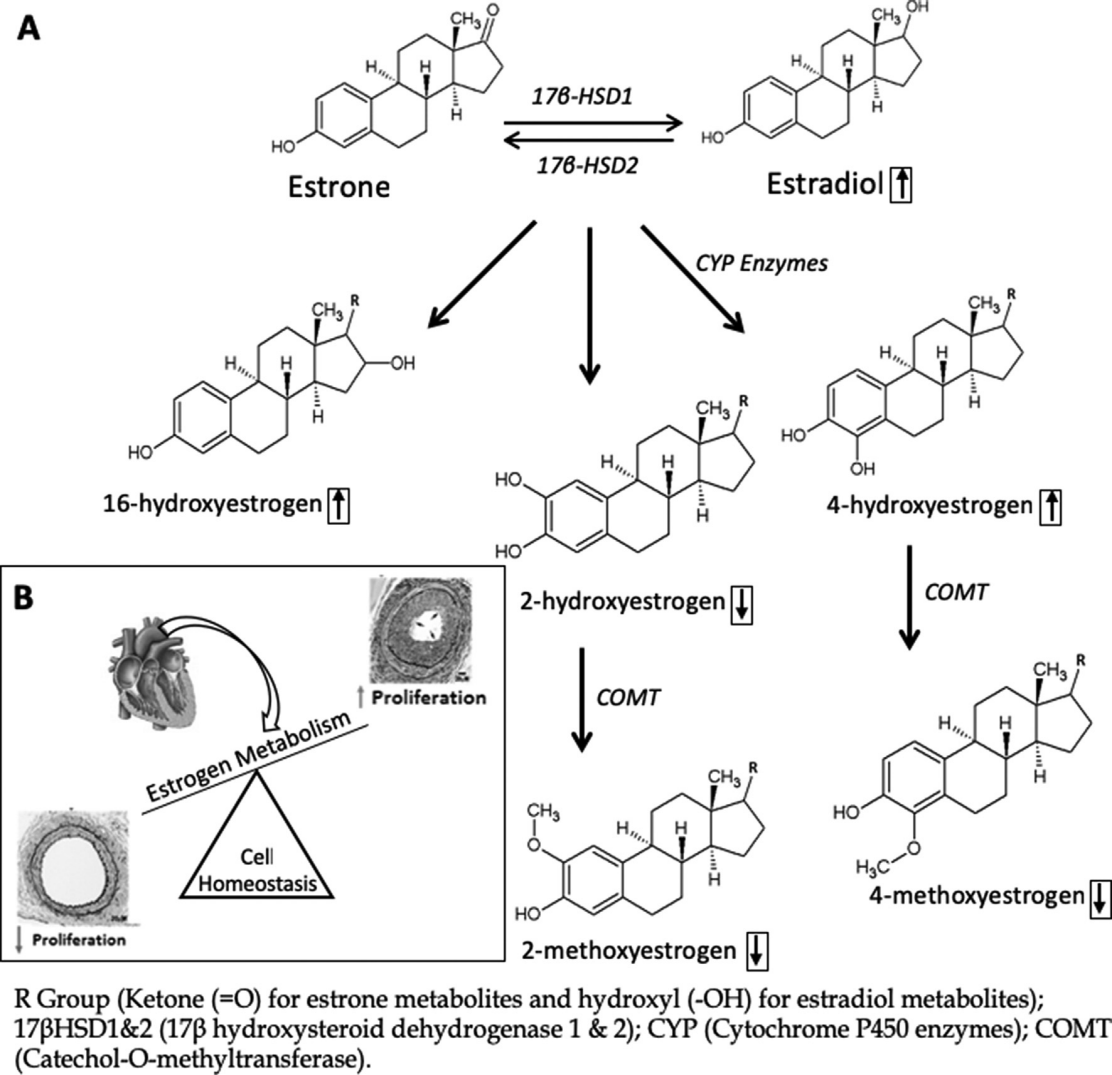
Estrogen metabolism; (A) The estrogen metabolism pathway, where parent estrogens, estrone and estradiol, are interchangeable by the action of 17β-HSDs prior to biotransformation by various cytochrome P450 enzymes inducing hydroxylation reactions in the 2′, 4′ or 16′ positions to form hydroxyestrogens. Rapid conversion/deactivation of the 2 and 4 hydroxylated estrogens then occurs via the action of COMT enzyme causing methylation in 2′ or 4′ positions. (B) Cardiovascular effects of imbalances in estrogen metabolism on normal cell homeostasis thought to drive both proliferative and antiproliferative cell phenotypes within the vasculature.

Estradiol and estrone circulate at low pico/femtomolar levels, ranging between 0.1 and 530 pg mL^−1^ (0.36–1945 pmol L^−1^) depending on age, sex and menstrual status [1,2]. They are metabolized by CYP450 enzymes, generating bioactive hydroxy and methoxy metabolites (Fig. 1A). The amounts of circulating downstream metabolites are assumed to be lower, but uncertainty exists due to a lack of accurate and precise methods for their measurement in blood [3]. The lack of a suitable analytical method hampers the investigation of several estrogen sensitive diseases such as breast cancer, uterine cancers and various cardiovascular diseases [4,5] where differential estrogen metabolism is thought to contribute [6,7] to mechanisms underpinning poor patient prognosis and outcomes [2]. A number of studies have demonstrated that imbalances in estrogen metabolism results in either proliferative or anti-proliferative effects in the vasculature (Fig. 1B) [8,9].

Current methods to quantify circulating levels of estrogens and their metabolites employ immunoassays such as ELISA, techniques limited by single analyte measurement, cross-reacting antibodies and batch variability. Therefore, a method to profile multiple estrogen metabolites simultaneously is in great demand. Liquid chromatography tandem mass spectrometry (LC-MS/MS) offers an alternative approach and is gold standard for analysis of steroids such as glucocorticoids and testosterone [10,11]. With estrogens being less abundant, methods are more challenging. Analysis of nascent estrogens has been reported [12] although derivatization is often required for LC-MS/MS. The major disadvantages associated with analyses of underivatized steroids are the extended extraction protocols employed to improve signal to noise and the large sample volumes required [4,5]. Derivatization improves sensitivity by addition of a permanently or readily-charged group, allowing higher ionization efficiency and increased sensitivity by MS. In turn this permits shorter extraction protocols and smaller sample volumes [14]. Derivatization methods commonly reported for analysis of E1 and E2 include use of dansyl chloride, *N*-methyl-nicotinic acid *N*-hydroxysuccinimide ester [15], 2-fluoro-1-methylpyridinium-p-toluenesulfonate [16] and isomers of 1,2-dimethylimidazole-sulfonyl chloride [17,18]. From these, dansyl chloride is most common [13,14,19,20], and its use has been extended to the downstream metabolites. However specificity of the assay for isobaric species is impeded since the product ions generated are identical for all estrogens, as they hail from the derivative [[19], [20], [21]]. Furthermore, dansylated products are light sensitive and the by-products of derivatization can interfere with analyses [22]. Therefore, alternative derivatization approaches should be explored.

Here we have validated a sensitive, specific and robust analytical method capable of detecting estrogens and their metabolites in human plasma. The method employed solid phase extraction (SPE) and methyl piperazine (MPPZ) derivatization, in conjunction with LC-MS/MS.

## 2 Materials and methods

### 2.1 Standards and solvents

Estrone (E1), 17β-estradiol (17βE2), 17α-estradiol (17αE2), 16-hydroxyestrone (16OHE1), 16-hydroxyestradiol (16OHE2), 2-methoxyestrone (2MeOE1), 4-methoxyestrone (4MeOE1), 2-methoxyestradiol (2MeOE2) and 4-methoxyestradiol (4MeOE2) were from Steraloids, Inc (Newport, USA). Internal standards 2,3,4–^13^C_3_-estrone (^13^C_3_-E1), 2,3,4–^13^C_3_-estradiol (^13^C_3_-E2), formic acid (FA) and methyl iodide (CH_3_I; ≥99%) were from Sigma-Aldrich, Inc. (St. Louis, USA). 2,3,4–^13^C_3_-16-hydroxyestradiol (^13^C_3_-16OHE2), 13,14,15,16,17,18-^13^C_6_-2-methoxyestrone (^13^C_6_-2MeOE1) and 13,14,15,16,17,18-^13^C_6_-4-methoxyestradiol (^13^C_6_-4MeOE2) were from CK Isotopes Limited (Leicestershire, UK). Certified estrone (1 mg mL^−1^ in MeOH; 1 mL) and 17β-estradiol (1 mg mL^−1^ in CH_3_CN; 1 mL) were from Cerilliant (Sigma Aldrich, Dorset, United Kingdom). 1-(5-Fluoro-2, 4-dinitrophenyl)-4-methylpiperazine (PPZ) was from TCI chemicals (Chuo-ku, Tokyo, Japan). HPLC grade solvents (methanol, acetone, acetonitrile and water) and LCMS grade (acetonitrile and water) solvents were from Fisher Scientific UK Limited (Leicestershire, UK).

### 2.2 Instrumentation

Analysis was performed on a QTrap 6500+ (Sciex, Warrington, UK) coupled to a Shimadzu Nexera X2 LC (Kyoto, Japan), operated using Analyst software v1.5.1.

### 2.3 Plasma samples

Female human plasma for method development and validation was from TCS Biosciences (Botolph Claydon, England), aliquots stored at −20 °C. This was prepared from blood of healthy human donors in approved blood collection centers. Additionally, we studied plasma from patients with pulmonary arterial hypertension (PAH) as it has previously been reported that estrogen levels are elevated in these patients and metabolites may be pathogenic [2,23,24]. PAH patient samples were collected with ethical approval within the Scottish Pulmonary Vascular Unit, Golden Jubilee National Hospital, Glasgow. The four female patients were as follows; 36 y idiopathic PAH (contraception unknown), 44 y; idiopathic PAH patient (Mirena^®^ coil), 46 y; idiopathic PAH (desogestrel) and a 55 y; hereditary PAH patient (tamoxifen).

### 2.4 Standard solutions

Estrogens (1 mg) and internal standards (IS; 1 mg) were dissolved in methanol (1 mL) and stored at −80 °C. Working solutions (0.0001–10 pg mL^−1^) were prepared by serial dilution on the day of use.

### 2.5 Generation of MPPZ derivatives

PPZ stock (10 μL; 1  mg mL^−1^), sodium bicarbonate (10 μL; 1M) and acetone (70 μL) were added to the standard/extract. The solution was incubated (60 °C, 1 h; Reaction 1). Reagents were reduced to dryness at 40 °C under oxygen free nitrogen (OFN). The dried residue was incubated (40 °C, 2 h; Reaction 2) with CH_3_I (100 μL). The mixture was reduced to dryness under OFN and dissolved in H_2_O/CH_3_CN (70:30; 70 μL).

### 2.6 Tuning and fragmentation analysis of MPPZ derivatives of estrogens and metabolites

The 6500 + Mass Spectrometer was operated in positive electrospray (ESI) mode as follows: curtain gas (40.0 psi), collision gas (medium), ion spray (5500 V), temperature (700 °C), nebulizer gas (60.0 psi) and heater gas (40.0 psi). For the detection of product ions by MS, molecular ions were isolated by their Q_1_ parent mass and subjected to collision induced dissociation (CID), in scanning mode using a range from *m/z* 450 to 640. Conditions for multiple reaction monitoring (MRM) were optimized by auto-tuning during infusion of the estrogen metabolites and IS (1 μg mL^−1^). The collision energy for each compound was optimized to achieve maximal sensitivity to detect quantifier and qualifier ions.

Structures of fragment ions formed from estrogen derivatives were determined by high resolution MS using a Synapt G2Si instrument (Waters Corp, Manchester, UK) fitted with an ESI source in positive mode. Samples (0.1 μg mL^−1^) dissolved in H_2_O: CH_3_CN (70:30) were infused 2 μL/min (Harvard Apparatus, UK) at a spray voltage 3.0 kV, sampling cone voltage 40 V and source temperature 100 °C. Data was collected in full scan mode and MS2 spectra (*m/z* 50 - 1200) in resolution mode. Tandem mass spectra were generated in the trapping region of the ion mobility cell using collision energy 40 V, with argon as the collision gas (40.0 psi). Instrument calibration was performed using 0.05M sodium formate. Lock mass correction was applied to precursor masses.

### 2.7 Chromatographic conditions

Estrogen metabolites were analyzed using an Ace Excel 2 C18-PFP column (150 × 2.1 mm 2 μm; HiChrom, Reading, England). A gradient solvent system of water: acetonitrile (90:10), containing FA (0.1%, 0.5 mL/min) was diverted to waste for the initial 9 min followed by elution for a further 4 min at 90:10, then with a gradient over 3 min until final conditions of water: acetonitrile (90:10) containing FA (0.1%, 0.5 mL/min) were achieved. Column and auto-sampler temperatures were 25 °C and 15 °C, respectively. Injection volume was 30 μL.

### 2.8 Extraction method

Aliquots of female plasma were subject to centrifugation (8000 g, 4 °C, 20 min) with the sediment discarded. Sample volumes (0.5 mL) were adjusted to 1 mL with water and enriched with internal standards (100 pg). Standard solutions were added into 1 mL water. SPE using Oasis^®^ MCX (3 cc/60 mg, Waters, Wilmslow, UK) extraction cartridges was performed under gravity. Prior to loading the sample, the cartridges were conditioned and equilibrated with methanol (2 mL), followed by water (2 mL). The diluted sample was loaded and allowed to pass through the cartridges and the eluate discarded. The cartridges were washed first with aqueous FA (2% v/v, 2 mL) and then with MeOH (30% v/v, 2 mL) and eluates discarded. Steroids were eluted in MeOH (100%; 2 mL). Extracts were reduced to dryness under OFN (40 °C) and the residues were derivatized as above.

### 2.9 Assay validation

#### 2.9.1 Optimization of derivatization conditions

Reaction conditions were optimized first using aqueous extracts and then using extracts of plasma. Conditions evaluated were incubation temperature (Reaction 1; 40–70 °C; Reaction 2; 40–60 °C), reaction time (Reaction 1; 30–90 min; Reaction 2; 30–180 min), reaction or reagent volume (Reaction 1; 10–30 μL, Reaction 2; 40–160 μL) and PPZ concentration (1–3 mg mL^−1^).

#### 2.9.2 Extraction efficiency

Recoveries of derivatives from water and plasma were assessed by comparison of mean peak areas of derivatives (1 ng; n = 6) following extraction, in pre-spiked samples divided by those in post-spiked samples.

#### 2.9.3 Matrix effects

Ion suppression of derivative signals in the presence of extracts of plasma was evaluated by comparing signal intensity of derivatives post-spiked into extracted plasma with that of aqueous estrogen solutions of the same concentration (1 ng; n = 6) following derivatization and expressed as a percentage.

#### 2.9.4 Specificity

Mass chromatograms were inspected at the retention times of estrogens and IS for interferences by other endogenous compounds in plasma.

#### 2.9.5 Limit of detection (LOD)

Estrogens (0.0005, 0.001, 0.002, 0.006, 0.01 ng and 0.5, 1, 2, 6, 10 pg mL^−1^) were analyzed by LC-MS/MS and the Signal/Noise (SNR) calculated. The limits of detection (LOD) were assigned at a SNR >3.

#### 2.9.6 Linearity

Blank samples and aliquots containing estrogens (1, 2, 6, 10, 20, 40, 100, 200, 500, 1000, 2000 pg mL^−1^) and internal standards (200 pg mL^−1^) were analyzed by LC-MS/MS. Calibration curves were plotted as the peak area ratio (standard/IS) versus amount of estrogens. Calibration lines were tested for lack of fit to a linear model and considered acceptable if the Fcalculated < Fcritical. Regression coefficients were deemed acceptable if r was >0.99. Weightings of equal, 1/x and 1/x^2^ were evaluated.

#### 2.9.7 Lower limit of quantitation (LLOQ)

Replicate aliquots (0.001, 0.002, 0.01 ng and 1, 2, 10 pg mL^−1^; n = 6) of estrogens and internal standards (0.2 ng; 200 pg mL^−1^) were analyzed as above. The LLOQ was calculated as that amount affording precision and accuracy of ∼20%.

#### 2.9.8 Accuracy and precision

The intra- and inter-day assay precision and accuracy were assessed using amounts of standard (2 & 200 or 2000 pg mL^−1^) and spiked plasma (100 pg mL^−1^ of metabolites only) prepared on the same day and different days, respectively (n = 6). The precision was calculated as the Relative Standard Deviation (RSD) (standard deviation/mean x 100), and % accuracy was the Relative Mean Error (RME), ((mean measured value - theoretical value)/theoretical value) x 100); precision and accuracy were accepted with RSD/RME <20% at LLOQ and <15% above. The bias of the calibration was tested against certified reference material for E1 and 17βE2; Bias, (Average Interday concentration – reference material concentration).

#### 2.9.9 Stability

Stability following storage in the auto-sampler (15 °C) and freezer (−20 and −80 °C) was evaluated by reinjection of a calibration standard and plasma sample (200 pg mL^−1^) after 24 h, 4, 8 and 31 days and expressed as % of the initial amount ((Sample Peak Area/T0 Peak Area) x 100).

### 2.10 Method application

The presence of estrogens and their metabolites was assessed, and their amounts quantified in human plasma from healthy female pooled controls (n = 3) and individual female PAH patients (n = 4) using the validated approach.

## 3 Result and discussion

### 3.1 Method development

#### 3.1.1 Development of derivatization approach

**Fig. 2:**
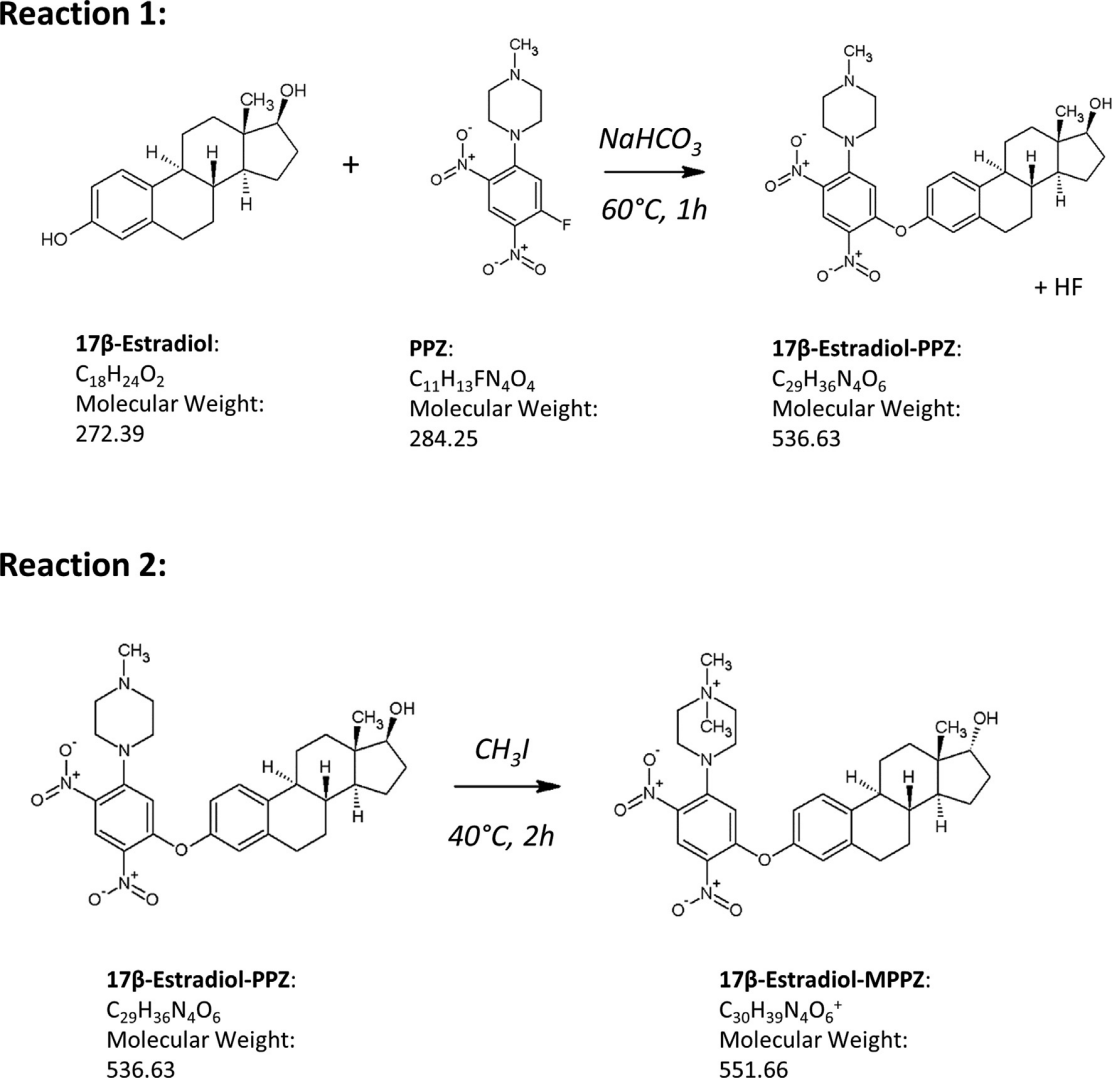
Formation of methylpiperazine (MPPZ) derivative of phenolic estrogens; showing an example of derivatization of 17β-estradiol with 1-(5-fluoro-2,4-dinitrophenyl)-4-methylpiperazine (PPZ) in the presence of sodium bicarbonate (NaHCO_3_) followed by reaction with methyl iodide (CH_3_I) to create the charged moiety.

All estrogen metabolites possess phenolic functional groups and are potential targets for derivatization by aromatic neutrophilic substitution, with additional ketone and/or hydroxyl groups present for estrone and estradiol metabolites, respectively. The phenolic group in the 3′ position of the A ring is most suitable for derivatization of the entire metabolite panel allowing clear mass distinction between steroids with ketones (estrone metabolites) and hydroxyls (estradiol metabolites) in the 17’ position of the D Ring. Fig. 2 illustrates the two-step MPPZ reaction with 17β-estradiol allowing formation of a permanently positively charged derivative. PPZ is a derivatization agent particularly well suited to steroid analysis by LC-ESI-MS/MS and has been applied to alternative matrices such as serum from pregnant women for commonly analyzed E1 and E2 but not for the bioactive metabolites [25]. It reacts rapidly and specifically with phenols to give 3-O-[2,4-dinitro-5-(4-methylpiperazino) phenylestrogens. A permanent positive charge is then appended by subsequent quaternarization of the piperazine amino group using methyl iodide [26]. Intense precursor and product ion signals in ESI-MS were observed, with the anticipated molecular ions of a singly-derivatized species shown in Table 1 which formed for all standards tested. 2 and 4 -hydroxy estrogens did not react well under these conditions and were explored separately [27]. Fragmentation patterns of the 9 derivatives (Table 1) were interrogated by accurate mass spectrometry (Supplementary Table 1) and putative structures assigned (Fig. 2), with masses within 10 Δppm of their theoretical values except in a few cases where signal intensity of fragments was low. Analyte specific product ions were assigned for EI and E2, however, certain metabolites generated similar fragments through the loss of the derivative function. Quantifier and qualifier ions were assigned (Table 1), but the common fragments produced by some of the metabolites led to an absolute need for chromatographic separation between isotopologues and isobars.

**Table 1:** **Liquid Chromatograpy- Tandem Mass Spectrometry:** Optimized tune parameters of methylpiperazine derivatives of estrogens and their retention times.

Analyte - MPPZ	Precursor Ion *m/z*	Product Ions *m/z* ^a^Quan ^b^Qual	Collision Energy (V)	Collision exit cell potential (V)	De-clustering potential (V)	Entrance potential (V)	Retention Time (mins)
E1	549.0	^a^502.3	59.0	20.0	141.0	10.0	15.5
		^b^72.0	99.0	12.0	131.0		
17αE2	551.0	^a^504.3	129.0	8.0	166.0	10.0	15.1
		^b^58.1	59.0	20.0	51.0		
17βE2	551.0	^a^504.3	129.0	8.0	166.0	10.0	14.6
		^b^58.1	59.0	20.0	51.0		
16OHE1	565.0	^a^58.0	105.0	10.0	130.0	10.0	13.2
		^b^251.4	59.0	10.0	130.0		
16OHE2	567.0	^a^58.0	121.0	8.0	136.0	10.0	12.1
		^b^251.0	61.0	22.0	166.0		
2MeOE1	579.0	^a^58.0	55.0	12.0	186.0	10.0	15.8
		^b^280.1	121.0	8.0	66.0		
4MeOE1	579.0	^a^280.1	121.0	8.0	66.0	10.0	15.6
		^b^58.0	55.0	12.0	186.0		
2MeOE2	581.0	^a^250.0	57.0	28.0	66.0	10.0	14.9
		^b^58.0	63.0	24.0	111.0		
4MeOE2	581.0	^a^250.0	57.0	28.0	66.0	10.0	14.6
		^b^58.0	63.0	24.0	111.0		
^13^C_3-_E1	552.3	^a^505.3	39.0	15.0	39.0	10.0	15.5
		^b^388.2	45.0	15.0	45.0		
^13^C_3-_E2	554.3	^a^507.3	35.0	15.0	35.0	10.0	14.6
		^b^390.3	40.0	15.0	40.0		
^13^C_3-_16OHE2	570.1	^a^58.1	100.0	15.0	135.0	10.0	12.1
		^b^72.10	100.0	15.0	135.0		
^13^C_6-_2MeOE1	585.0	^a^58.1	100.0	15.0	125.0	10.0	15.8
		^b^280.1	100.0	15.0	50.0		
^13^C_6-_4MeOE2	587.0	^a^58.1	100.0	15.0	100.0	10.0	14.6
		^b^280.00	100.0	15.0	75.0		

Voltage (V); Estrone (E1); estradiol (17 α/β E2); 16-hydroxyestrone (16OHE1); 16-hydroxyestradiol (16OHE2); 2 or 4-methoxyestrone (2 or 4-MeOE1); 2 or 4-methoxyestradiol (2 or 4-MeOE2); 2,3,4–^13^C_3_-estrone (^13^C_3^-^_E1); 2,3,4–^13^C_3_-estradiol (^13^C_3_-E2); 2,3,4–^13^C_3_-estriol (^13^C_3_-16OHE2); 13,14,15,16,17,18-^13^C_6_-2-methoxyestrone (^13^C_6_-2MeOE1) and 13,14,15,16,17,18-^13^C_6_-4-methoxyestradiol (^13^C_6_-4MeOE2).

**Fig. 3:**
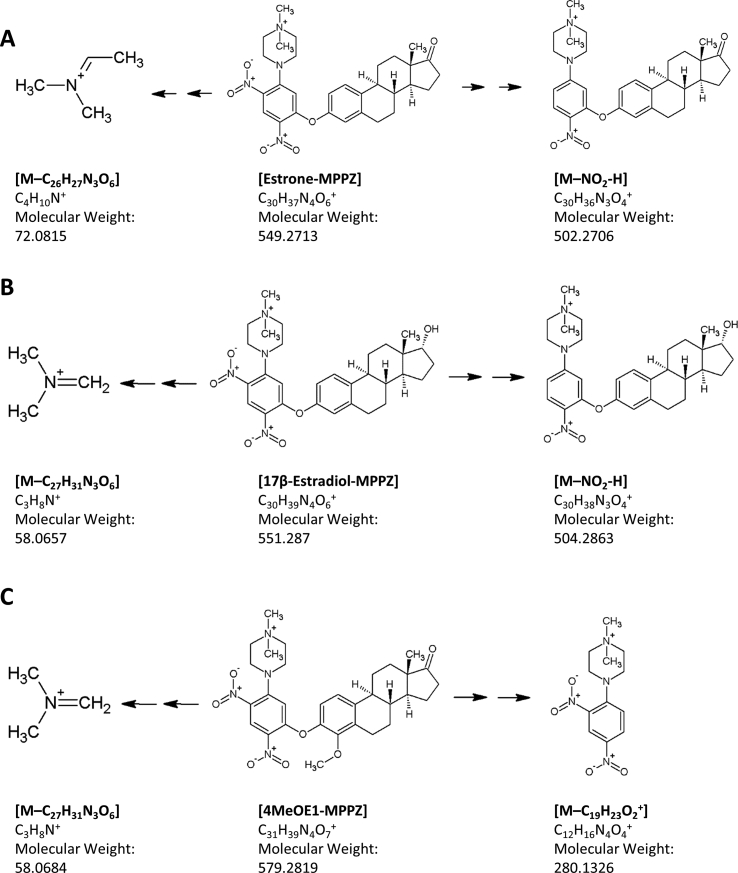
Proposed fragmentation patterns following accurate mass analysis at 1 μg mL^−1^ of estrogen methylpiperazine derivatives displaying common mass fragments; (A) Estrone (B) 17β- Estradiol (C) 4MeOEl.

The MPPZ derivatives of E1 and 17βE2 fragmented to give two specific product ions *m/z* 549 → 502 and 551 → 504 (Fig. 3) by loss of NO_2_. These masses demonstrated an increment of M+3 for their stably labelled counterparts at *m/z* 505.3 and *m/z* 507.3, respectively, supporting the presence of the steroidal A ring. Smaller fragments of *m/z* 72 and *m/z* 58 were also formed, lacking stable isotope functions and common across several of the metabolite derivatives. These result through loss of [M-C_4_H_10_N^+^] and [M-C_3_H_8_N^+^] respectively from the methylated piperazine structure (Fig. 2, Supplementary Table 1). For methoxyestrogens, product ions of *m/z* 280 and 250 were observed, again without mass increments in the labelled species and thus less specific for analytes. The difference of unit mass between the fragment ions of *m/z* 250/251 and *m/z* 280/281 between methoxy and hydroxy metabolites respectively suggests a block to proton migration from the 2′ or 4’ position of estrogen due to the methylated group.

#### 3.1.2 Optimization of derivatization reaction conditions

The efficiency of the initial PPZ reaction was not improved by alterations in reaction time, temperature or by reagent volume, concentration and pH. In the second methylation reaction, the efficiency and reproducibility of derivative formation was improved by increasing reaction time (30–120 min) and decreasing the reaction temperature (60–40 °C). Additionally, reconstitution of samples in mobile phase of higher organic proportions (70:30 H_2_O:CH_3_CN) improved signal intensities. Additionally, it should be noted that the use of plastic 96-well microtitre plates for the analysis of MPPZ-estrogen derivatives is not recommended as when used inconsistent signal responses for spiked samples were observed and derivative stability was poorer.

#### 3.1.3 Chromatographic conditions

Chromatographic conditions were assessed on five LC columns representing a variety of stationary phases (Acquity Atlantis T3, ACE Ultra Core Super C18, ACE Excel Super C18, ACE Excel C18-AR and ACE Excel C18-PFP, all 150 mm in length). Separation could not be achieved on the Acquity Atlantis T3 contained C18 groups on particle sizes of 3 μm, whereas the small particles of 2.5 μm in the ACE Ultra Core Super C18 allowed partial but incomplete peak separation. The ACE Excel Super C18 with its unique encapsulated bonding technology and ACE Excel C18-AR with its integrated phenyl capacity chosen for added selectivity for aromatic compounds still did not resolve the methoxyestrogen metabolites. The ACE Excel C18 with integral pentafluorophenyl functionality added further selectivity via its π-π interactions and was the only column able to successfully resolve the methoxyestrogen metabolites alongside the wider panel, with typical retention times shown in Table 1.

#### 3.1.4 Extraction

Liquid-liquid extraction of plasma has been applied to recover estrogens, e.g. using dichloromethane or ethyl acetate, however manual liquid extraction can lead to imprecision, with cumbersome protocols and lower recovery rates [28,29]. Extraction of estrogen and metabolites using Oasis^®^ MCX separation columns with the manufacturer's generic protocol allowed high recovery (93–108%) of the 9 target metabolites (Table 2). However, significant ion suppression was identified when the method was applied to plasma; signals were typical suppressed to by 56–71%, even following the 2% FA wash. Ion suppression was minimized (Table 2) by washing with 30% MeOH prior to elution with 100% MeOH. Alternate conditions for 16αOHE1 and 16αOHE2 applying a 40% MeOH wash and 95% MeOH elution reduced ion suppression further (18 ± 18% and 22 ± 10%, respectively). This change however negatively influenced the quality of data for the other metabolites and was not pursued for use with the whole panel.

**Table 2:** **Extraction efficiency, limits of quantitation and linearity of response**. Assessment of the extraction efficiency of estrogens from plasma showing high recovery rates and decreased ion suppression upon optimization. Calibration curves over a wide linear range are presented, and the limits of quantitation.

Analyte	IS	Recovery (%)	Generic IonS (%)	Optimized IonS (%)	LLOD pg mL^−1^ (pg on column)	LLOQ pg mL^−1^ (pg on column)	ULOQ pg mL^−1^ (pg on column)	Mean r
E1	^13^C_3_E1	108 ± 9%	- 63 ± 11%	- 9 ± 19%	1 (0.21)	2 (0.43)	2000 (434.7)	0.998
17αE2	^13^C_3_E2	101 ± 11%	- 62 ± 11%	- 6 ± 18%	2 (0.43)	2 (0.43)	2000 (434.7)	0.990
17βE2	^13^C_3_E2	102 ± 10%	- 71 ± 8%	+ 17 ± 8%	1 (0.21)	2 (0.43)	2000 (434.7)	0.993
16OHE1	^13^C_3_-16OHE2	111 ± 10%	- 62 ± 8%	- 37 ± 6%	2 (0.43)	10 (2.17)	2000 (434.7)	0.998
16OHE2	^13^C_3_-16OHE2	103 ± 14%	- 69 ± 5%	- 40 ± 8%	2 (0.43)	10 (2.17)	2000 (434.7)	0.996
2MeOE1	^13^C_6_-2MeOE1	98 ± 10%	- 56 ± 11%	- 3 ± 16%	6 (1.30)	10 (2.17)	2000 (434.7)	0.996
4MeOE1	^13^C_6_-2MeOE1	102 ± 16%	- 56 ± 5%	- 11 ± 15%	6 (1.30)	10 (2.17)	2000 (434.7)	0.996
2MeOE2	^13^C_6_-4MeOE2	93 ± 11%	- 59 ± 4%	- 9 ± 13%	6 (1.30)	10 (2.17)	2000 (434.7)	0.995
4MeOE2	^13^C_6_-4MeOE2	98 ± 15%	- 58 ± 8%	- 14 ± 19%	6 (1.30)	10 (2.17)	2000 (434.7)	0.996

Estrone (E1); estradiol (17 α/β E2); 2, 4 or 16-hydroxyestrone (16OHE1); 16-hydroxyestradiol (16OHE2); 2 or 4-methoxyestrone (2 or 4-MeOE1); 2 or 4-methoxyestradiol (2 or 4-MeOE2); IS = Internal Standard; Recovery = Prespike/pospiked estrogen; IonS = Ion Suppression = Postspike derivatized estrogen/unextracted peak areas; Generic = Manufacturer's Protocol; Optimized = addition of MeOH wash step; LLOD/Q = lower limit of detection/quantitation; ULOQ = Upper level of quantitation; pg mL^-1^ (pg on column).

### 3.2 Assay validation

Validation of the panel of 9 estrogens was progressed.

#### 3.2.1 Specificity

**Fig. 4:**
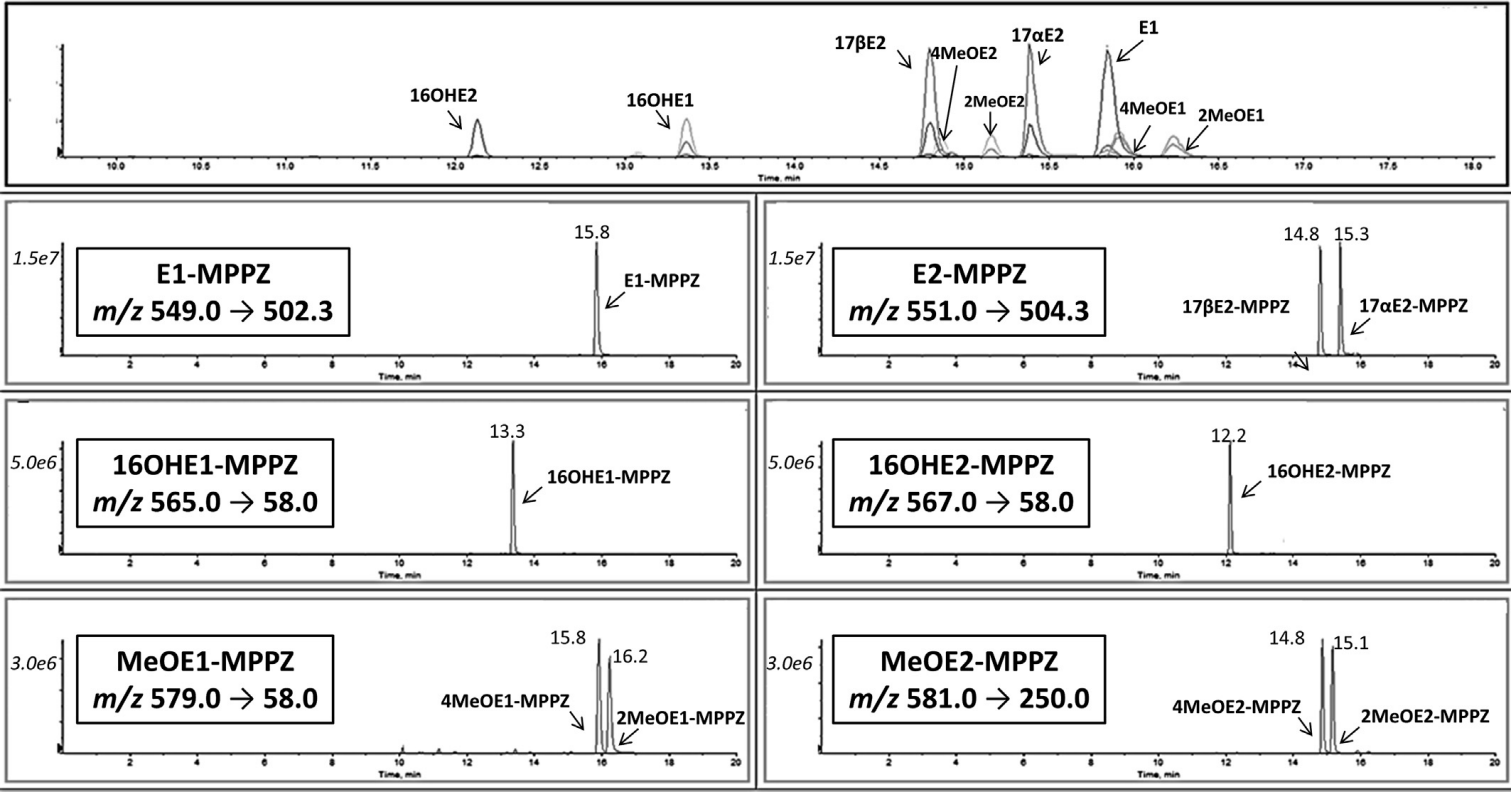
Mass chromatograms of methylpiperazine (MPPZ) derivatives of estrogens and their metabolites following analysis of an extracted solution of Standards, 2000 pg mL^-1^. Total Ion Chromatograms and the corresponding extracted ion chromatograms showing resolution of derivatives of estrone (El), estradiol (17α & 17βE2), 16- hydroxyestrogens (16OHE1 & 16OHE2) and methoxyestrogens (2MeOEl, 4MeOEl, 2MeOE2 & 4MeOE2) by retention time and mass transition.

Baseline chromatographic separation of estrogen derivatives was achieved in aqueous standards. When applied to plasma samples, interferences closely eluting at the retention time of each estrogen were not observed, such as 17α-E2, which displays a different retention time to 17βE2 (Fig. 4).

#### 3.2.2 Linearity

Stable-isotope labelled estrogens (^13^C_3_ and ^13^C_6_) were selected as internal standards, dependent on availability (Table 2). One labelled estrogen per chemical grouping was chosen due to cost constraints but paired internal standards are available for all analytes and are recommended. Derivatives quantified with exact labelled versions for comparison demonstrated a more accurate response (Table 3). A mean r-value > 0.99 for calibration lines was achieved for all derivatives with a weighting of 1/x throughout. A linear relationship was also confirmed as curves also passed lack of fit tests (Supplemental Table 2). The linear range achieved is similar to that of methods quantifying E1 and E2 using other sensitive derivatives developed in the past few years for both plasma [16,30] and urinary analysis [31].

#### 3.2.3 Limits of detection and quantitation

The LOD and LLOQs for estrogen MPPZ derivatives (Table 2) measured in standard solutions are comparable to previous methods for the analysis of E1 and E2 [16,21,[32], [33], [34]]. The volume of plasma (0.5 mL) extracted by SPE for detection of metabolites demonstrated an improvement over recent methods for oncology studies; one study by Yang et al., 2008 used 2 mL of patient serum using a longer extraction protocol where samples were incubated overnight prior to liquid extraction of estrogens [35]. Another study applied dansyl chloride and achieved low LLOQs of 0.05–0.1 pg on column but required specialized equipment not commonly available within hospitals and clinical laboratories [36].

#### 3.2.4 Precision and accuracy

The values for intra- and inter-assay precision and accuracy (Table 3) were acceptable at low points of the calibration curve (<20%) 0.43 & 2.17 pg on column (2 and 10 pg mL^−1^) and <15% above this level; 43.4 & 434.0 pg on column (200 or 2000 pg mL ^−1^). Accuracy of calibration data compared favorably with certified reference material for E1 and E2 (metabolites not available; Supplemental Table 3). The bias was higher at ULOQ when a weighting of 1/x was applied, this could be corrected by removing this weighting to achieve a better fit at higher concentrations. In general, we expect the quantified levels to fall below this point. In addition, acceptable precision was shown for endogenous E1 and E2 and for the six metabolites enriched in healthy female plasma samples (100 pg mL^−1^ metabolites), except for 4MeOE1. The upward deviation may have reflected presence of low endogenous levels of this species.

**Table 3:** **Intra-day and inter-day validation**. Summary table of the accuracy and precision data showing acceptable intra-day and inter-day reproducibility. For standards, criteria were met at the LLOQ and ULOQ. For metabolites the mid concentration is shown rather than ULOQ, as this is more relevant to expected levels. For plasma, validation was performed in aliquots containing endogenous estrone and estradiol and enriched with metabolites.

Metabolite	Target (pg mL^−1^)	Intraday (n = 6)			Interday (n = 6)		
		Mean (pg mL^−1^)	Precision (RSD %)	Accuracy (RME %)	Mean (pg mL^−1^)	Precision (RSD %)	Accuracy (RME %)
E1	2	2.2	8.7	7.4	2.0	8.0	0.3
	2000	2464	14.0	14.1	2232	13.9	6.1
	Endogenous	37	13.3	–	31	13.1	–
17αE2	2	2.1	10.6	5.0	1.9	12.1	1.6
	2000	2210	11.2	10.6	2280	6.7	13.9
	Endogenous	ND	–	–	ND	–	–
17βE2	2	2.1	7.6	4.2	2.0	7.6	3.6
	2000	2244	11.5	6.1	1864	12.1	5.3
	Endogenous	24	13	–	28	12.4	–
16OHE1	10	9.3	3.9	7.4	10.0	1.4	1.8
	200	228	9.4	14.4	196	9.7	1.7
	Plasma + 100	96	6.9	4.8	101	11.8	1.2
16OHE2	10	9.7	5.0	3.2	10.1	1.5	1.8
	200	202	7.4	7.4	194	9.9	3.4
	Plasma + 100	112	4.1	12	104	4.2	4.6
2MeOE1	10	9.4	10.0	4.1	10.2	9.4	1.5
	200	176	10.6	12.2	203	5.6	3.4
	Plasma + 100	114	4.1	12.0	104	6.3	2.7
4MeOE1	10	10.1	2.7	11.8	9.7	5.7	1.2
	200	220	13.3	8.6	202	6.9	1.6
	Plasma + 100	108	11.5	7.0	120	14.2	20.7
2MeOE2	10	8.8	4.2	11.7	9.5	7.2	5.2
	200	198	14.3	0.8	204	4.1	2.4
	Plasma + 100	108	11.7	7.7	108	14.4	8.5
4MeOE2	10	9.0	5.6	8.8	10.3	2.5	2.6
	200	198	8.9	1.0	228	5.9	6.6
	Plasma + 100	104	4.8	3.2	106	9.6	6.6

Estrone (E1); estradiol (17 α/β E2); 16-hydroxyestrone (16 OHE1); 16-hydroxyestradiol (16 OHE2); 2 or 4-methoxyestrone (2 or 4-MeOE1); 2 or 4-methoxyestradiol (2 or 4-MeOE2); RSD, standard deviation/mean x 100, RME %, Relative Mean Error ((mean measured value - theoretical value)/theoretical value x 100); Endogenous levels in female plasma; Plasma +100 (metabolites spiked in plasma at 100 pg mL^−1^). ND = not detected.

#### 3.2.5 Stability

The MPPZ derivatives in extracts of standards and plasma demonstrated suitable stability upon short term storage up to 8 days in the auto sampler (15 °C) and upon longer term storage of 31 days in the freezer (−20 °C), with less than 20% degradation. Storage at lower temperatures (−80 °C) caused degradation of methoxyestrogens over the 31 days (50% or upon rethaw), but was suitable for E1, E2 and 16-OHE1/2 metabolites (Supplementary Table 4).

### 3.3 Method application

**Fig. 5:**
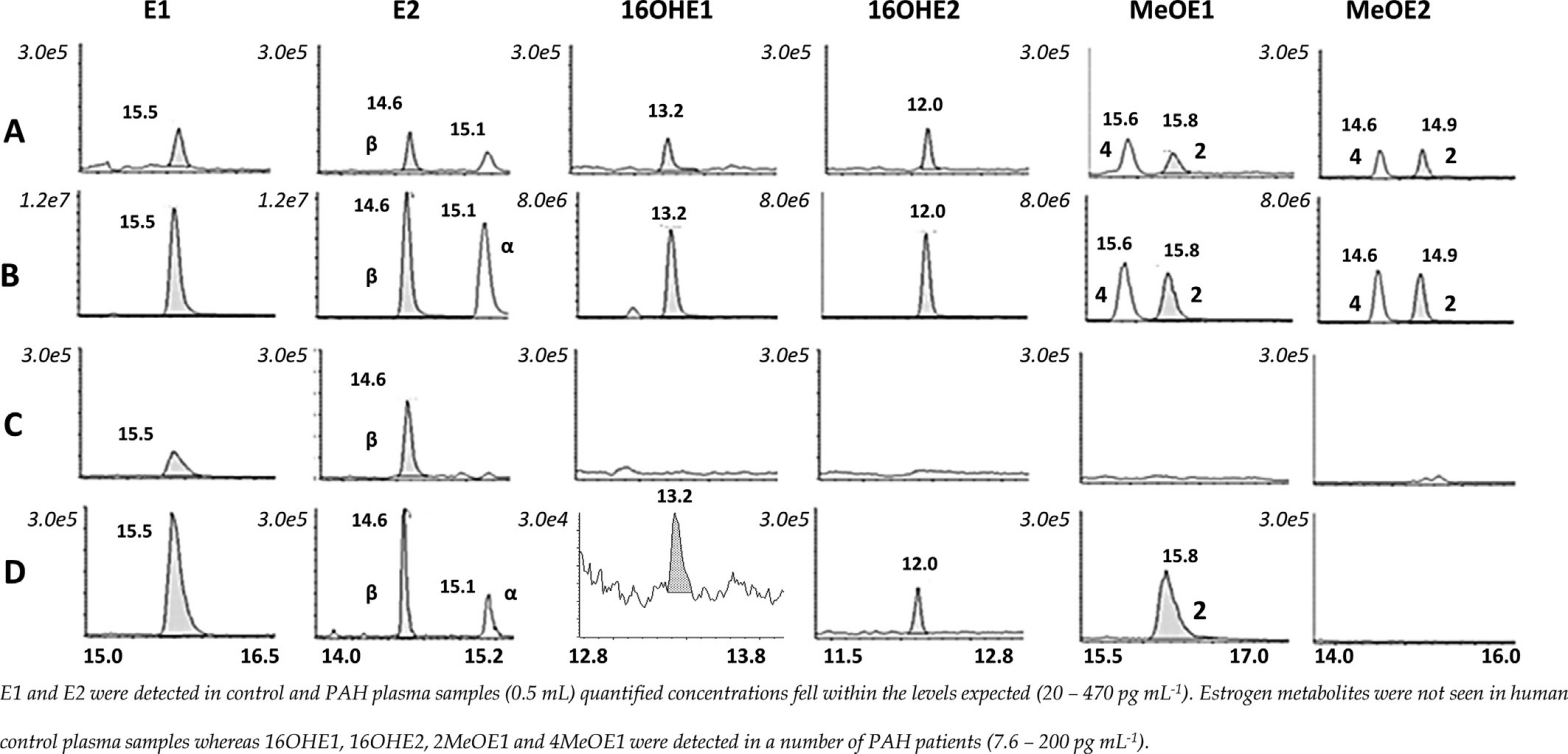
Mass chromatograms of methylpiperazine (MPPZ) derivatives of estrone (E1), estradiol (17α & 17βE2), 16-hydroxyestrogens (16OHE1 & 16OHE2) and methoxyestrogens (2MeOE1, 4MeOE1, 2MeOE2 & 4MeOE2) extracted from plasma. Extracted ion chromatograms of derivatized estrogens at (A) the lower (2 or 10 pg mL^−1^), and (B) upper limit of quantitation (2000 pg mL^−1^) and in plasma (C) from control female subjects and (D) female patients with Pulmonary Arterial Hypertension (PAH). El and E2 wcre detected in control and PAH plasma (0.5 mL) and concentrations fell within the levels expected (20–470 pg mL^−1^). Estrogen metabolites were not detected in human control plasma samples, whereas 16OHE1,16OHE2,2MeOEl and 4MeOE1 were detected in a number of PAH patients (7.6–200 pg mL^−1^).

The method was applied to samples from female pooled controls vs female PAH patient plasma. Calculated concentrations of estrone and estradiol were compared to reported biological concentrations and fall within the expected ranges (E1/17βE2 = 73.4 – 1725.3 pmol L^−1^) whereas metabolites were only detected in PAH patient plasma (27.8 - 734.2 pmol L^-1^) (Fig. 5), albeit only small numbers of samples were analyzed.

## 4 Conclusion

In summary, derivatization of estrone, estradiol and six of its bioactive metabolites by MPPZ derivatization allows quantification to desired levels in patient samples. This approach for plasma analysis compares favorably with several LC-MS/MS methods developed for E1 and E2 with comparable sensitivities and an improved stability of derivatives. Derivatization of this extended panel of estrogen metabolites for their simultaneous analysis in plasma has not been available routinely in the past for clinical analyses. This approach offers a novel wider view of estrogen metabolism, which appears highly relevant to estrogen sensitive diseases and will now be applied to larger cohort studies.

## Appendix A Supplementary data

Denver et al-supplemental Denver et al-supplemental
