# Supplementary material for: Derivatization enhances analysis of estrogens and their bioactive metabolites in human plasma by liquid chromatography tandem mass spectrometry
Source: Anal Chim Acta. 2019 Apr 25;1054:84–94. doi: 10.1016/j.aca.2018.12.023 (PMC6363983; doi:10.1016/j.aca.2018.12.023)
Supplement: Denver et al-supplemental [file mmc1.docx]

Derivatization enhances analysis of estrogens and their bioactive metabolites in human plasma by liquid chromatography tandem mass spectrometry

Nina Denver^a,b^, Shazia Khan^a^, Ioannis Stasinopoulos^a^, Colin Church^c^, Natalie ZM Homer^a^, Margaret R MacLean^b^, Ruth Andrew^a^

^a^ Mass Spectrometry Core, Edinburgh Clinical Research Facility, Queen’s Medical Research Institute, 47 Little France Crescent, Edinburgh, EH16 4TJ, United Kingdom

^b^ Institute of Cardiovascular and Medical Sciences, College of Medical, Veterinary and Life Sciences, University of Glasgow, University Avenue, Glasgow, G12 8QQ, United Kingdom

^c^ Scottish Pulmonary Vascular unit, Golden Jubilee National Hospital, Agamemnon St, Clydebank, G81 4DY, United Kingdom

Author for correspondence: Professor Ruth Andrew

Email address: [ruth.andrew@ed.ac.uk](mailto:ruth.andrew@ed.ac.uk), postal address as above

Telephone: +44-131-242-6763

Email address of all authors: [n.denver.1@research.gla.ac.uk](mailto:n.denver.1@research.gla.ac.uk), [Shazia.Khan@ed.ac.uk](mailto:Shazia.Khan@ed.ac.uk), [s1474874@sms.ed.ac.uk](mailto:s1474874@sms.ed.ac.uk), [colinchurch@nhs.net](mailto:colinchurch@nhs.net), [n.z.m.homer@ed.ac.uk](mailto:n.z.m.homer@ed.ac.uk), and Mandy.MacLean@glasgow.ac.uk

**Supplemental Table 1:** High-resolution mass spectrometric analysis of estrogen-MPPZ derivatives and putative structural identification of product ions

| **Analyte-MPPZ** | **Accurate Mass Precursor Ion** | **Molecular Formula Precursor Ion** | **Theoretical Product Ion**  **Mass** | **Observed Product Ion Mass** | **Proposed Product Ion Molecular Formula** | **Product Ion Δ**  **ppm** |
| --- | --- | --- | --- | --- | --- | --- |
| E1 | 549.2713 | C_30_H_37_N_4_O_6_ | ^a^502.2705  ^b^72.0813 | ^a^502.2706  ^b^72.0815 | C_30_H_36_N_3_O_4_  C_4_H_10_N | 0.04  2.77 |
| 17αE2 | 551.2870 | C_30_H_39_N_4_O_6_ | ^a^504.2862  ^b^58.0656 | ^a^504.2863  ^b^58.0657 | C_30_H_38_N_3_O_4_  C_3_H_8_N | 0.19  1.72 |
| 17βE2 | 551.2870 | C_30_H_39_N_4_O_6_ | ^a^504.2862  ^b^58.0656 | ^a^504.2863  ^b^58.0657 | C_30_H_38_N_3_O_4_  C_3_H_8_N | 0.19  1.72 |
| 16OHE1 | 565.2662 | C_30_H_37_N_4_O_7_ | ^a^58.0656  ^b^250.1191 | ^a^58.0657  ^b^250.1184 | C_3_H_8_N  C_12_H_16_N_3_O_3_ | 1.72  2.79 |
| 16OHE2 | 567.2819 | C_30_H_39_N_4_O_7_ | ^a^58.0656  ^b^250.1191 | ^a^58.0655  ^b^250.1187 | C_3_H_8_N  C_12_H_16_N_3_O_3_ | 1.72  1.59 |
| 2MeOE1 | 579.2819 | C_31_H_39_N_4_O_7_ | ^a^58.0656  ^b^280.1171 | ^a^58.0661  ^b^280.1170 | C_3_H_8_N  C_12_H_16_N_4_O_4_ | 8.61  0.35 |
| 4MeOE1 | 579.2819 | C_31_H_39_N_4_O_7_ | ^a^280.1171  ^b^58.0656 | ^a^280.1209  ^b^58.0652 | C_12_H_16_N_4_O_4_  C_3_H_8_N | 13.56*  6.88 |
| 2MeOE2 | 581.2975 | C_31_H_41_N_4_O_7_ | ^a^250.1191  ^b^58.0656 | ^a^250.1176  ^b^58.0639 | C_12_H_16_N_3_O_3_  C_3_H_8_N | 5.99  29.28* |
| 4MeOE2 | 581.2975 | C_31_H_41_N_4_O_7_ | ^a^250.1191  ^b^58.0656 | ^a^250.1190  ^b^58.0652 | C_12_H_16_N_3_O_3_  C_3_H_8_N | 0.39  6.88 |
| ^13^C_3_E1 | 552.2814 | ^13^C_3_C_27_H_37_N_4_O_6_ | ^a^505.2806  ^b^388.2017 | ^a^505.2806  ^b^388.2017 | ^13^C_3_C_27_H_36_N_3_O_4_  ^13^C_3_C_22_H_25_N_2_O_2_ | 0.00  0.00 |
| ^13^C_3_E2 | 554.297 | ^13^C_3_C_27_H_39_N_4_O_6_ | ^a^507.2963  ^b^390.2173 | ^a^507.2970  ^b^390.2183 | ^13^C_3_C_27_H_38_N_3_O_4_  ^13^C_3_C_22_H_27_N_2_O_2_ | 1.37  2.56 |
| ^13^C_3_16αOHE2 | 570.2919 | ^13^C_3_C_27_H_39_N_4_O_7_ | ^a^58.0656  ^b^72.0813 | ^a^58.0655  ^b^72.0812 | C_3_H_8_N  C_4_H_10_N | 1.72  1.39 |
| ^13^C_6_2MeOE1 | 585.3020 | ^13^C_6_C_25_H_39_N_4_O_7_ | ^a^58.0656  ^b^280.1171 | ^a^58.0661  ^b^280.1163 | C_3_H_8_N  C_12_H_16_N_4_O_4_ | 8.61  2.85 |
| ^13^C_6_4MeOE2 | 587.3177 | ^13^C_6_C_25_H_41_N_4_O_7_ | ^a^58.0656  ^b^280.1171 | ^a^58.0655  ^b^280.1163 | C_3_H_8_N  C_12_H_16_N_4_O_4_ | 1.72  2.85 |

*Fragments with low signal intensity following infusion generated higher ppm values.

Voltage (V); Estrone (E1); estradiol (17 α/ β E2); 16-hydroxyestrone (16 OHE1); 16-hydroxyestradiol (16 OHE2); 2 or 4-methoxyestrone (2 or 4-MeOE1); 2 or 4-methoxyestadiol (2 or 4-MeOE2); 2,3,4-^13^C_3_-estrone (^13^C_3_E1); 2,3,4- ^13^C_3_-estradiol (^13^C_3_E2); ; 2,3,4-^13^C_3_-estriol (^13^C_3_-16αOHE2); 13,14,15,16,17,18-^13^C_6_-2-methoxyestrone (^13^C_6_-2MeOE1) and 13,14,15,16,17,18-^13^C_6_-4-methoxyestradiol (^13^C_6_-4MeOE1)

**Supplemental Table 2:** Lack-of-fit calibration analysis of residual variance

|  | **E1 & E2** | **Metabolites** |
| --- | --- | --- |
| **Number of samples (N)** | 8 | 9 |
| **Power (P)** | 6 | 6 |
| **DoF (numerator, n-2)** | 7 | 6 |
| **DoF (denominator, n*(p-1)** | 5 | 40 |
| **Analyte-MPPZ** | **F(Calculated)** |  |
| **E1** | 0.08 |  |
| **17αE2** | 0.07 |  |
| **17βE2** | 0.08 |  |
| **Fcritical** | 2.22 |  |
| **16OHE1** | 0.13 |  |
| **16OHE2** | 0.10 |  |
| **2MeOE1** | 0.08 |  |
| **4MeOE1** | 0.09 |  |
| **2MeOE2** | 0.21 |  |
| **4MeOE2** | 0.09 |  |
| **Fcritical** | 3.33 |  |

If Fcalc > Fcrit then reject the null hypothesis, If Fcalc > Fcrit then reject the null hypothesis; As Fcalc < Fcrit the null hypothesis is accepted meaning there is no lack of fit in these calibration curves; DoF = degrees of freedom; MSS = Mean Sum of squares; LOF = Lack of fit; Fcalc = MSS (LOF)/MSS (error)

**Supplemental Table 3:** Comparison of standards to certified reference material for E1 and 17βE2, determination of assay bias

|  |  | **1/X weighting** | | | **No weighting** | | |
| --- | --- | --- | --- | --- | --- | --- | --- |
| **E1**  **pg mL^-1^** | **C_ref_** | **C_average_** | **Bias** | **%** | **C_average_** | **Bias** | **%** |
| **2** | **2.4** | **2.2** | **-0.2** | **-0.2** | **-** | **-** | **-** |
| **20** | **22.2** | **20.4** | **-1.8** | **-1.8** | **-** | **-** | **-** |
| **100** | **104.6** | **102.6** | **-2.0** | **-2** | **-** | **-** | **-** |
| **200** | **199.7** | **186.3** | **-13.4** | **-13.4** | **203.7** | **3.9** | **1.9** |
| **2000** | **2038.8** | **2464** | **425.2** | **425.2** | **2206.59** | **167.8** | **8.2** |
| **Plasma** | **30.4** | **31.4** | **1.0** | **1** | **-** | **-** | **-** |
|  |  | **1/X weighting** | | | **No weighting** | | |
| **E2**  **pg mL^-1^** | **C_ref_** | **C_average_** | **Bias** | **%** | **C_average_** | **Bias** | **%** |
| **2** | **2.1** | **2.1** | **0.0** | **0.0** | **-** | **-** | **-** |
| **20** | **19.0** | **18** | **-1.0** | **-5.6** | **-** | **-** | **-** |
| **100** | **98.7** | **114.8** | **16.1** | **14.0** | **-** | **-** | **-** |
| **200** | **196.3** | **202.2** | **5.9** | **2.9** | **201.7** | **5.4** | **2.7** |
| **2000** | **2092.4** | **2244** | **151.6** | **6.8** | **2052.4** | **-39.9** | **-1.9** |
| **Plasma** | **29.5** | **27.9** | **-1.6** | **-1.6** | **-** | **-** | **-** |

Estrone (E1); estradiol (17βE2); C_average_, average result from laboratory; Cr_ef_, reference material; Bias = C_average_ - C_ref_; Bias (%) = (C_average_ - C_ref_/C_average_)*100

**Supplemental Table 4: Stability of estrogen-MPPZ derivatives in standard and plasma extracts following storage;** short term for 1, 4 and 8 days in the Autosampler at 15°C and longer term for 4, 8 and 31 days in the freezer at -20°C and -80°C

| **STANDARDS** | **Autosampler (15°C)** | | | **-20°C** | | | **-80°C** | | |
| --- | --- | --- | --- | --- | --- | --- | --- | --- | --- |
| **Days** | ***1*** | ***4*** | ***8*** | ***4*** | ***8*** | ***31*** | ***4*** | ***8*** | ***31*** |
| E1 | 84 | 86 | 88 | 115 | 103 | 114 | 94 | 119 | 112 |
| 17αE2 | 88 | 82 | 89 | 102 | 100 | 75 | 86 | 114 | 67 |
| 17βE2 | 92 | 81 | 86 | 107 | 94 | 111 | 77 | 104 | 88 |
| 16αOHE1 | 93 | 95 | 80 | 90 | 79 | 117 | 91 | 85 | 109 |
| 16αOHE2 | 91 | 106 | 97 | 87 | 76 | 75 | 54 | 84 | 77 |
| 2MeOE1 | 81 | 90 | 85 | 111 | 89 | 103 | 58 | 98 | 96 |
| 4MeOE1 | 83 | 91 | 91 | 109 | 100 | 76 | 97 | 118 | 76 |
| 2MeOE2 | 102 | 93 | 104 | 115 | 108 | 114 | 86 | 119 | 101 |
| 4MeOE2 | 108 | 94 | 104 | 121 | 105 | 108 | 82 | 111 | 83 |

| **PLASMA** | **Autosampler (15°C)** | | | **-20°C** | | | **-80°C** | | |
| --- | --- | --- | --- | --- | --- | --- | --- | --- | --- |
| **Days** | ***1*** | ***4*** | ***8*** | ***4*** | ***8*** | ***31*** | ***4*** | ***8*** | ***31*** |
| E1 | 104 | 140 | 109 | 129 | 111 | 123 | 123 | 122 | 98 |
| 17αE2 | 72 | 95 | 92 | 97 | 94 | 71 | 92 | 99 | 58 |
| 17βE2 | 85 | 104 | 94 | 107 | 91 | 104 | 102 | 100 | 76 |
| 16αOHE1 | 72 | 102 | 91 | 99 | 98 | 87 | 102 | 98 | 81 |
| 16αOHE2 | 108 | 138 | 120 | 116 | 112 | 82 | 116 | 119 | 85 |
| 2MeOE1 | 76 | 106 | 93 | 92 | 86 | 85 | 103 | 99 | 51 |
| 4MeOE1 | 111 | 148 | 119 | 133 | 117 | 94 | 123 | 119 | 75 |
| 2MeOE2 | 81 | 105 | 102 | 105 | 93 | 84 | 105 | 109 | 49 |
| 4MeOE2 | 83 | 109 | 105 | 106 | 94 | 87 | 103 | 106 | 55 |

All values expressed as a percentage of the original peak area response at T=0 hours ((T=0 peak area/ T= days peak area)*100)
